# Supplementary material for: The Evolution and Characterization of the RNA Interference Pathways in Lophotrochozoa
Source: Genome Biol Evol. 2024 May 7;16(5):evae098. doi: 10.1093/gbe/evae098 (PMC11114477; doi:10.1093/gbe/evae098)
Supplement: evae098_Supplementary_Data [file evae098_supplementary_data.zip › Supplementary_materials.zip!/selected_miRNAs/NoGe_selected_miRNAs/mirdeep_pdf_files/NMRB01000470.1_Notospermus_geniculatus_strain_Ushimado_scaffold470,_whole_genome_shotgun_sequence_14115.pdf]

Provisional ID	:	NMRB01000470.1_Notospermus_geniculatus_strain_Ushimado_scaffold470,_whole_genome_shotgun_sequence_14115
Score total	:	29200.6
Score for star read(s)	:	3.9
Score for read counts	:	29192.2
Score for mfe	:	1.5
Score for randfold	:	0
Score for cons. seed	:	3
Total read count	:	57271
Mature read count	:	57255
Loop read count	:	0
Star read count	:	16

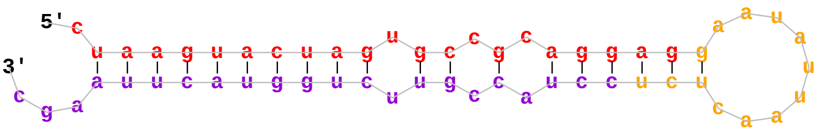

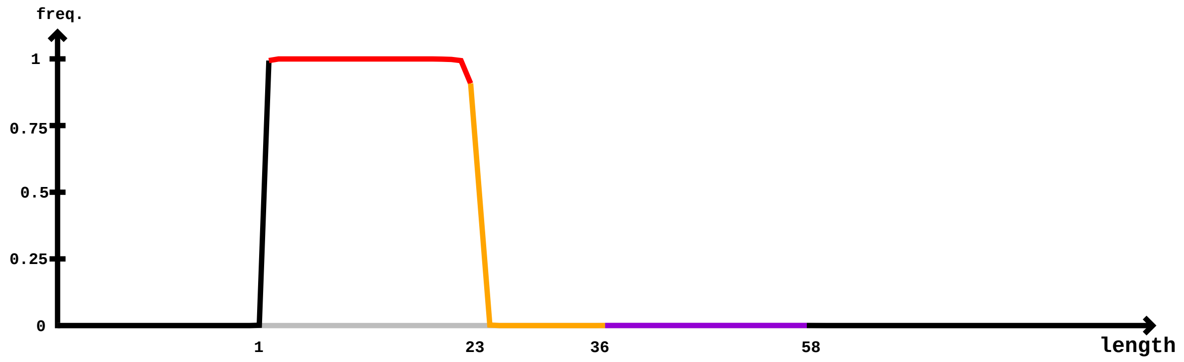


```
                    Mature                          Star
5'- augagagccaucgucagauacuaaguacuagugccgcaggaggaauauuaacucuccuaccguucugguacuuaagcuaggacauggcgaacacaucucuggagggagcuga  -3'   obs
    augagagccaucgucagauacuaaguacuagugccgcaggaggaauauuaacucuccuaccguucugguacuuaagcuaggacauggcgaacacaucucuggagggagcuga        exp
    ......(((((.(((......((((((((((.((.(.((((((.........)))))).).)).))))))))))......)))))))).....((.((((....)))).)).  reads   mm   sample
    ...................acuaaguacuagugccgcagga.......................................................................  1       0    Mm1
    ....................cuaaguacuagugccgca..........................................................................  5       0    Mm1
    ....................cuaaguacuagugccgcag.........................................................................  9       0    Mm1
    ....................cuaaguacuagugccgcagU........................................................................  9       1    Mm1
    ....................cuaaguacuagugccgcagg........................................................................  1       0    Mm1
    ....................cuaaguacuagugccgcUgga.......................................................................  1       1    Mm1
    ....................cuaaguacuagugccgcaggU.......................................................................  14      1    Mm1
    ....................cuaaguUcuagugccgcagga.......................................................................  1       1    Mm1
    ....................cuaaguacuagugccgcagga.......................................................................  293     0    Mm1
    ....................cuaaguacuagugccgcGgga.......................................................................  1       1    Mm1
    ....................cuaaguacuagugccgcCgga.......................................................................  3       1    Mm1
    ....................cuaaguacuagugccgAagga.......................................................................  2       1    Mm1
    ....................cuaaguacuaUugccgcagga.......................................................................  1       1    Mm1
    ....................cuaaguacuagugccgcaggaC......................................................................  9       1    Mm1
    ....................cuaagAacuagugccgcaggag......................................................................  1       1    Mm1
    ....................cuaaguacuagugccgUaggag......................................................................  6       1    Mm1
    ....................cuaaguacuagugccgAaggag......................................................................  13      1    Mm1
    ....................cuaaguacuagugccgcGggag......................................................................  14      1    Mm1
    ....................cuaaguacuagugccgcUggag......................................................................  9       1    Mm1
    ....................cAaaguacuagugccgcaggag......................................................................  1       1    Mm1
    ....................Nuaaguacuagugccgcaggag......................................................................  3       1    Mm1
    ....................cuaaguacuagugccgcaggaA......................................................................  15      1    Mm1
    ....................cuaaguacuagugccgcaUgag......................................................................  1       1    Mm1
    ....................cuaaguacuagugAcgcaggag......................................................................  1       1    Mm1
    ....................cuaaguacuagugccgGaggag......................................................................  2       1    Mm1
    ....................cuaaguacuagugccgcaggag......................................................................  2957    0    Mm1
    ....................cuCaguacuagugccgcaggag......................................................................  1       1    Mm1
    ....................Uuaaguacuagugccgcaggag......................................................................  5       1    Mm1
    ....................cuaaguacuaCugccgcaggag......................................................................  2       1    Mm1
    ....................cuaaguUcuagugccgcaggag......................................................................  1       1    Mm1
    ....................cuUaguacuagugccgcaggag......................................................................  1       1    Mm1
    ....................cuaaguacuagugccgcCggag......................................................................  8       1    Mm1
    ....................cuaaguacuaAugccgcaggag......................................................................  5       1    Mm1
```

```
                    Mature                             Star
augagagccaucgucagauacuaaguacuagugccgcaggaggaauauuaacucuccuaccguucugguacuuaagcuaggacauggcgaacacaucucuggagggagcuga
....................cuaaguacuaUugccgcaggag......................................................................   1      1   Mm1
....................cuaCguacuagugccgcaggag......................................................................   2      1   Mm1
....................cuaaguCcuagugccgcaggag......................................................................   1      1   Mm1
....................cCaaguacuagugccgcaggag......................................................................   1      1   Mm1
....................cuaaguacuagugccgcaggaU......................................................................   11     1   Mm1
....................cuaaguGcuagugccgcaggag......................................................................   1      1   Mm1
....................cuaaguacuagugccgcaggagA.....................................................................   30     1   Mm1
....................cuaaguacuagugccgcaggagC.....................................................................   2      1   Mm1
....................cuaaguacuagugccgcaggagU.....................................................................   5899   1   Mm1
....................cuaaguacuagugccgcaggagg.....................................................................   33     0   Mm1
....................cuaaguacuagugccgcaggaggU....................................................................   16     1   Mm1
.....................uaaguacuagugccgcaggag......................................................................   9      0   Mm1
.....................uaaguacuagugccgcaggagU.....................................................................   30     1   Mm1

...................acuaaguacuagugccgcagga.......................................................................   5      0   Mm2
....................cuaaguacuagugccgca..........................................................................   3      0   Mm2
....................cuaaguacuagugccgcaU.........................................................................   3      1   Mm2
....................Uuaaguacuagugccgcagg........................................................................   1      1   Mm2
....................cuaaguacuagugccgcagC........................................................................   1      1   Mm2
....................cuaaguacuagugccgcagg........................................................................   50     0   Mm2
....................cuaaguacuagugccgcagU........................................................................   2      1   Mm2
....................cAaaguacuagugccgcagga.......................................................................   1      1   Mm2
....................cuaaguacuagugccgcaggG.......................................................................   10     1   Mm2
....................cuaaguacuagugccgcCgga.......................................................................   2      1   Mm2
....................cuaaguacuagugccgUagga.......................................................................   2      1   Mm2
....................cCaaguacuagugccgcagga.......................................................................   1      1   Mm2
....................cuaaguacuaguCccgcagga.......................................................................   1      1   Mm2
....................cuaaguacuagugccgcUgga.......................................................................   4      1   Mm2
....................cuaaguacuaCugccgcagga.......................................................................   1      1   Mm2
....................cuaaguacuagugccgAagga.......................................................................   3      1   Mm2
....................cuaaguacuagugccgcagga.......................................................................   887    0   Mm2
....................cuaaguacuagugccgcaggU.......................................................................   13     1   Mm2
....................cuaaguacuagugccgcGgga.......................................................................   4      1   Mm2
....................cuaaguUcuagugccgcagga.......................................................................   1      1   Mm2
....................cuaaguacuagugccgGagga.......................................................................   2      1   Mm2
....................cuaaguacuagugccgAaggag......................................................................   26     1   Mm2
....................cuaaguacuagugccgcaUgag......................................................................   1      1   Mm2
....................cGaaguacuagugccgcaggag......................................................................   1      1   Mm2
....................cuUaguacuagugccgcaggag......................................................................   1      1   Mm2
....................cuaaguacuaAugccgcaggag......................................................................   7      1   Mm2
....................cuaaguUcuagugccgcaggag......................................................................   4      1   Mm2
....................cuaagCacuagugccgcaggag......................................................................   2      1   Mm2
....................cuaaguacuagugccgcaggaA......................................................................   3      1   Mm2
....................cuGaguacuagugccgcaggag......................................................................   2      1   Mm2
....................cuaaguacuagugccgUaggag......................................................................   16     1   Mm2
....................cuaaguacuagugccgcCggag......................................................................   13     1   Mm2
....................cAaaguacuagugccgcaggag......................................................................   1      1   Mm2
....................cuaaguacuaUugccgcaggag......................................................................   6      1   Mm2
....................cuaUguacuagugccgcaggag......................................................................   2      1   Mm2
....................cuaCguacuagugccgcaggag......................................................................   1      1   Mm2
....................cuaaguacuagugcGgcaggag......................................................................   2      1   Mm2
....................cuaaguacuUgugccgcaggag......................................................................   2      1   Mm2
....................cuaaguacuagugccgcUggag......................................................................   20     1   Mm2
....................cuaaguacuagugccgcaggUg......................................................................   2      1   Mm2
....................cuaaguacuaCugccgcaggag......................................................................   8      1   Mm2
....................cuaaguacuagugccgGaggag......................................................................   7      1   Mm2
....................Nuaaguacuagugccgcaggag......................................................................   2      1   Mm2
....................cuaagAacuagugccgcaggag......................................................................   1      1   Mm2
....................cuaaguacuagugcUgcaggag......................................................................   1      1   Mm2
....................cuaaguacuagugccgcGggag......................................................................   38     1   Mm2
....................cuaaguacuagugccgcaggCg......................................................................   1      1   Mm2
....................cuaaguacuagugccgcaggaU......................................................................   80     1   Mm2
....................cuaaguacuagugccgcaggag......................................................................   6372   0   Mm2
....................cuaaguacuagugccgcaggaC......................................................................   59     1   Mm2
....................cuaaguacuagugAcgcaggag......................................................................   2      1   Mm2
....................Uuaaguacuagugccgcaggag......................................................................   11     1   Mm2
....................cuaaguacuagugccgcaggGg......................................................................   3      1   Mm2
....................cuaaguacuagCgccgcaggag......................................................................   1      1   Mm2
....................cuaaguacuaCugccgcaggagg.....................................................................   1      1   Mm2
....................cuaaguacuagugccgcaggagU.....................................................................   1826   1   Mm2
```

Mature **Star**

```
augagagccaucgucagauacuaaguacuagugccgcaggaggaauauuaacucuccuaccguucugguacuuaagcuaggacauggcgaacacaucucuggagggagcuga
....................cuaaguacuagugccgcaggaCg.....................................................................   1     1   Mm2
....................cuaaguacuagugccgcUggagg.....................................................................   1     1   Mm2
....................cuaaguacuagugccgUaggagg.....................................................................   1     1   Mm2
....................cuaaguacuagugccgcaggagg.....................................................................   139   0   Mm2
....................cuaaguacuagugccgcaggagA.....................................................................   142   1   Mm2
....................cuaaguacuagugccgcaggaggU....................................................................   7     1   Mm2
....................cuaaguacuagugccgcaggagAa....................................................................   1     1   Mm2
.....................uaaguacuagugccgcagga.......................................................................   7     0   Mm2
.....................uaaguacuagugccgcaggag......................................................................   33    0   Mm2
.....................uaaguacuagugccgcaggaU......................................................................   1     1   Mm2
.....................uaaguacuagugccgcaggagU.....................................................................   21    1   Mm2
.....................uaaguacuagugccgcaggagg.....................................................................   20    0   Mm2
.......................aguacuagugccgcaggag......................................................................   1     0   Mm2
.......................................................ccuaccguucugguacuuaagc...................................   1     0   Mm2

....................cuaaguacuagugccgca..........................................................................   6     0   Fm2
....................cuaaguacuagugccgcaA.........................................................................   5     1   Fm2
....................cuaaguacuCgugccgcagg........................................................................   5     1   Fm2
....................cuaaguacuagugccgcagg........................................................................   32    0   Fm2
....................cuaaguacuagugccgcGgga.......................................................................   1     1   Fm2
....................cuaaguacuagugccgcCgga.......................................................................   1     1   Fm2
....................cuaaguacuaCugccgcagga.......................................................................   1     1   Fm2
....................cuaaguacuagugccgcaggG.......................................................................   2     1   Fm2
....................Nuaaguacuagugccgcagga.......................................................................   1     1   Fm2
....................Auaaguacuagugccgcagga.......................................................................   1     1   Fm2
....................cuaaguacuagugccgcagUa.......................................................................   1     1   Fm2
....................cuaaguacuaUugccgcagga.......................................................................   3     1   Fm2
....................cuaaguacuagugccgcagga.......................................................................   292   0   Fm2
....................cuaaguacuUgugccgcagga.......................................................................   1     1   Fm2
....................cuaaguacuagugccgcUgga.......................................................................   1     1   Fm2
....................cuaaguacuagugccgUaggag......................................................................   4     1   Fm2
....................cuaaguacuagugccgcUggag......................................................................   11    1   Fm2
....................cuaaguacuagugccgcagUag......................................................................   1     1   Fm2
....................cuaaguacAagugccgcaggag......................................................................   1     1   Fm2
....................cuaaguacuagugcUgcaggag......................................................................   2     1   Fm2
....................cuaaguacuagugccgGaggag......................................................................   2     1   Fm2
....................cuaaguacuagugccgcaggaA......................................................................   6     1   Fm2
....................cNaaguacuagugccgcaggag......................................................................   1     1   Fm2
....................cuaaguacuagugccgcaggag......................................................................   3042  0   Fm2
....................cuaaguacuagugccgcCggag......................................................................   8     1   Fm2
....................cuaaguacuagugccgcaggaU......................................................................   19    1   Fm2
....................cuaaguNcuagugccgcaggag......................................................................   2     1   Fm2
....................cCaaguacuagugccgcaggag......................................................................   1     1   Fm2
....................cuaaguacuaUugccgcaggag......................................................................   4     1   Fm2
....................cuaaguacuaAugccgcaggag......................................................................   1     1   Fm2
....................cuaaguacuagCgccgcaggag......................................................................   1     1   Fm2
....................cuaCguacuagugccgcaggag......................................................................   1     1   Fm2
....................Auaaguacuagugccgcaggag......................................................................   2     1   Fm2
....................cuaaguacuaCugccgcaggag......................................................................   4     1   Fm2
....................Uuaaguacuagugccgcaggag......................................................................   4     1   Fm2
....................cuaagCacuagugccgcaggag......................................................................   1     1   Fm2
....................cuaaguacuagugccgcaUgag......................................................................   1     1   Fm2
....................cuaaguacuagugccgcGggag......................................................................   8     1   Fm2
....................cuaaguacuagugccgcagAag......................................................................   1     1   Fm2
....................Nuaaguacuagugccgcaggag......................................................................   1     1   Fm2
....................cuaaguacuagugccgAaggag......................................................................   12    1   Fm2
....................cuaaguacuagAgccgcaggag......................................................................   1     1   Fm2
....................cuaaguacuagugccgcaggaC......................................................................   44    1   Fm2
....................cAaaguacuagugccgcaggag......................................................................   1     1   Fm2
....................cuaaguacuagugccgcaggagg.....................................................................   92    0   Fm2
....................cuaaguacuagugccgcaggagA.....................................................................   42    1   Fm2
....................cuaaguacuagugccgcaggagU.....................................................................   3900  1   Fm2
....................cuaaguacuagugccgcaggagC.....................................................................   4     1   Fm2
....................cuaaguacuagugccgcCggagg.....................................................................   2     1   Fm2
....................cuaaguacuagugccgcaggaggG....................................................................   1     1   Fm2
....................cuaaguacuagugccgcaggaggU....................................................................   19    1   Fm2
.....................uaaguacuagugccgcaggag......................................................................   4     0   Fm2
.....................uaaguacuagugccgcaggagU.....................................................................   10    1   Fm2

...................acuaaguacuagugccgcagga.......................................................................   25    0   Fm1
```

Mature **Star**

```
augagagccaucgucagauacuaaguacuagugccgcaggaggaauauuaacucuccuaccguucugguacuuaagcuaggacauggcgaacacaucucuggagggagcuga
...................acuaaguacuagugccUcagga.......................................................................	1	1	Fm1
...................acuaaguacuagugccgcaggaU......................................................................	11	1	Fm1
....................cuaaguacuagugccgca..........................................................................	6	0	Fm1
....................cuaaguacuagugccgcU..........................................................................	2	1	Fm1
....................cuaaguacuagugccgcCg.........................................................................	1	1	Fm1
....................cuaaguacuagugccgcaU.........................................................................	4	1	Fm1
....................cuaaguacuagugccgcag.........................................................................	6	0	Fm1
....................cuaaguacuagugccgcUgg........................................................................	1	1	Fm1
....................cuaaguacuagugccgGagg........................................................................	1	1	Fm1
....................cuaaguacuagugccgcagU........................................................................	6	1	Fm1
....................cuaaguacuagugccgcagg........................................................................	24	0	Fm1
....................Nuaaguacuagugccgcagga.......................................................................	1	1	Fm1
....................cuaaguacuaCugccgcagga.......................................................................	1	1	Fm1
....................cuaaguacuagugccgcagga.......................................................................	1331	0	Fm1
....................cuaaguacuagugccgUagga.......................................................................	2	1	Fm1
....................cuaaguacuagugUcgcagga.......................................................................	2	1	Fm1
....................cAaaguacuagugccgcagga.......................................................................	3	1	Fm1
....................Uuaaguacuagugccgcagga.......................................................................	1	1	Fm1
....................cuGaguacuagugccgcagga.......................................................................	2	1	Fm1
....................cuaaguacuagugccgcaggG.......................................................................	7	1	Fm1
....................cuaaguacuagugccgGagga.......................................................................	1	1	Fm1
....................cuaaguacuagugccAcagga.......................................................................	1	1	Fm1
....................cuaaguacuagugccgcUgga.......................................................................	5	1	Fm1
....................cuaaguacuaAugccgcagga.......................................................................	1	1	Fm1
....................cuaaguGcuagugccgcagga.......................................................................	1	1	Fm1
....................cuaaguacuagugccgAagga.......................................................................	4	1	Fm1
....................cuaaguacuagugccgcCgga.......................................................................	1	1	Fm1
....................cuaaguacuagugccgcaggU.......................................................................	11	1	Fm1
....................cuaaguacuagugccgcGgga.......................................................................	7	1	Fm1
....................cuaaguacuagugccgAaggag......................................................................	15	1	Fm1
....................cuaaguUcuagugccgcaggag......................................................................	2	1	Fm1
....................cuaaguacuagugccgcaggaU......................................................................	87	1	Fm1
....................cuaaguacuUgugccgcaggag......................................................................	1	1	Fm1
....................cuaaguGcuagugccgcaggag......................................................................	1	1	Fm1
....................cuaaguacuagugccgGaggag......................................................................	4	1	Fm1
....................cuaaguacuagugccgcUggag......................................................................	9	1	Fm1
....................cuaaguacuagugcUgcaggag......................................................................	2	1	Fm1
....................cuGaguacuagugccgcaggag......................................................................	1	1	Fm1
....................cuaaguacuagugccgUaggag......................................................................	3	1	Fm1
....................cuaaguacuagGgccgcaggag......................................................................	1	1	Fm1
....................cuaagGacuagugccgcaggag......................................................................	1	1	Fm1
....................cuaaguacuagugccgcaUgag......................................................................	1	1	Fm1
....................cuaaguacuagugccgcaggaC......................................................................	37	1	Fm1
....................cCaaguacuagugccgcaggag......................................................................	2	1	Fm1
....................cuaaguacuagugccgcaggUg......................................................................	1	1	Fm1
....................cuaaguacuagugccgcaggag......................................................................	3405	0	Fm1
....................Uuaaguacuagugccgcaggag......................................................................	5	1	Fm1
....................cuaaguacuagugccgcGggag......................................................................	13	1	Fm1
....................cuaaguacuaUugccgcaggag......................................................................	4	1	Fm1
....................cAaaguacuagugccgcaggag......................................................................	1	1	Fm1
....................cuaaguacuaAugccgcaggag......................................................................	9	1	Fm1
....................cuaaguacuaCugccgcaggag......................................................................	2	1	Fm1
....................cuaaguacuaguUccgcaggag......................................................................	1	1	Fm1
....................cuaaguacuagugAcgcaggag......................................................................	1	1	Fm1
....................cuaaguaUuagugccgcaggag......................................................................	2	1	Fm1
....................cuaaguacuagugccgcagUag......................................................................	2	1	Fm1
....................Nuaaguacuagugccgcaggag......................................................................	2	1	Fm1
....................cuaaguacuaguAccgcaggag......................................................................	1	1	Fm1
....................cuaaguacuagugccgcCggag......................................................................	20	1	Fm1
....................cuaaguNcuagugccgcaggag......................................................................	1	1	Fm1
....................cuaaguacuagugccgcaggaA......................................................................	3	1	Fm1
....................cuaaguacuagCgccgcaggag......................................................................	2	1	Fm1
....................cuaaguacuagAgccgcaggag......................................................................	1	1	Fm1
....................cuaaguacuagugccgcaggagC.....................................................................	11	1	Fm1
....................cuaaguacuagugccgAaggagg.....................................................................	3	1	Fm1
....................cuaaguacuagugccgcaggagA.....................................................................	219	1	Fm1
....................cuaaguacuagugccgcaggagg.....................................................................	135	0	Fm1
....................cuaaguacuagugccgcCggagg.....................................................................	2	1	Fm1
....................cuaaguacuagugccgcaggagU.....................................................................	718	1	Fm1
....................cuaaguacuagugccgcaggaCg.....................................................................	1	1	Fm1
```

```
                    Mature                             Star
augagagccaucgucagauacuaaguacuagugccgcaggaggaauauuaacucuccuaccguucugguacuuaagcuaggacauggcgaacacaucucuggagggagcuga
....................cuaaguacuagugccgcaggaggU....................................................................    14    1    Fm1
.....................uaaguacuagugccgcagg........................................................................    2     0    Fm1
.....................uaaguacuagGgccgcagga.......................................................................    1     1    Fm1
.....................uaaguacuagugccgcaCga.......................................................................    1     1    Fm1
.....................uaaguacuagugccgcagga.......................................................................    14    0    Fm1
.....................uaaguacuagugccgcCggag......................................................................    1     1    Fm1
.....................uaaguacuagugccgcaggag......................................................................    16    0    Fm1
.....................uaaguacuagugccgcaggagg.....................................................................    3     0    Fm1
.....................uaaguacuagugccgcaggagU.....................................................................    2     1    Fm1
.......................................................ccuaccguucugguacuuaagc...................................    5     0    Fm1

...................acuaaguacuagugccgcagg........................................................................    2     0    Mm3
...................acuaaguacuagugccgcagga.......................................................................    2     0    Mm3
...................acuaaguacuagugccgcaggag......................................................................    1     0    Mm3
...................acuaaguacuagugccgcaggaU......................................................................    1     1    Mm3
...................Ccuaaguacuagugccgcaggag......................................................................    1     1    Mm3
...................acuaaguacuagugccgcaggagU.....................................................................    1     1    Mm3
....................cuaaguacuagugccgca..........................................................................    1     0    Mm3
....................cuaaguacuagugccgcag.........................................................................    8     0    Mm3
....................cuaaguacuagugccgcagU........................................................................    1     1    Mm3
....................cuaaguacuagugccgcagg........................................................................    51    0    Mm3
....................cuaaguacuagugccgUagg........................................................................    1     1    Mm3
....................cuaaAuacuagugccgcagg........................................................................    1     1    Mm3
....................cuaaguacuagugccgcaggG.......................................................................    3     1    Mm3
....................cuaaguacuagugccgGagga.......................................................................    1     1    Mm3
....................cuaaAuacuagugccgcagga.......................................................................    1     1    Mm3
....................Nuaaguacuagugccgcagga.......................................................................    1     1    Mm3
....................cuaaguacuagugccgcagga.......................................................................    590   0    Mm3
....................cuaaguacuagugccgcCgga.......................................................................    2     1    Mm3
....................cuaaguacuaUugccgcagga.......................................................................    1     1    Mm3
....................cuaaguacuagugccgcaggU.......................................................................    13    1    Mm3
....................cuaaguacuagugccgcUgga.......................................................................    2     1    Mm3
....................cuaaguacuaAugccgcagga.......................................................................    1     1    Mm3
....................Uuaaguacuagugccgcagga.......................................................................    1     1    Mm3
....................cuaaguacuaguAccgcagga.......................................................................    1     1    Mm3
....................cuaaguacuagugccgcaggC.......................................................................    2     1    Mm3
....................cuaaguacuagugccgcaggag......................................................................    6598  0    Mm3
....................cuaaguacuagugccgGaggag......................................................................    5     1    Mm3
....................cuUaguacuagugccgcaggag......................................................................    1     1    Mm3
....................cuGaguacuagugccgcaggag......................................................................    6     1    Mm3
....................cuaaguacuagAgccgcaggag......................................................................    1     1    Mm3
....................cuaaguacuaUugccgcaggag......................................................................    7     1    Mm3
....................cuaaguacuagugccgcaggCg......................................................................    3     1    Mm3
....................cuaCguacuagugccgcaggag......................................................................    1     1    Mm3
....................cuaaguacAagugccgcaggag......................................................................    1     1    Mm3
....................cuaagCacuagugccgcaggag......................................................................    1     1    Mm3
....................cuaaguacuaCugccgcaggag......................................................................    4     1    Mm3
....................cuaagAacuagugccgcaggag......................................................................    2     1    Mm3
....................Nuaaguacuagugccgcaggag......................................................................    6     1    Mm3
....................cuaaguacuUgugccgcaggag......................................................................    2     1    Mm3
....................cuaaguacuagugccgcaggaU......................................................................    61    1    Mm3
....................cuaaguacuagugccgcaAgag......................................................................    1     1    Mm3
....................cuaaguacuagugccgcaggUg......................................................................    1     1    Mm3
....................cuaaguaAuagugccgcaggag......................................................................    1     1    Mm3
....................Uuaaguacuagugccgcaggag......................................................................    13    1    Mm3
....................cuaaguacuagugccgcUggag......................................................................    20    1    Mm3
....................cuaaguacuaAugccgcaggag......................................................................    5     1    Mm3
....................cuaaguacuagugccgAaggag......................................................................    29    1    Mm3
....................cuaaguUcuagugccgcaggag......................................................................    1     1    Mm3
....................cuaaguacuagugccgcaggaA......................................................................    10    1    Mm3
....................cuaaguacuagugccgcCggag......................................................................    16    1    Mm3
....................cuaaguacuaguCccgcaggag......................................................................    1     1    Mm3
....................cuaaguacuagugGcgcaggag......................................................................    1     1    Mm3
....................cuaaguacuagugccgUaggag......................................................................    14    1    Mm3
....................cuaaguacuagugccAcaggag......................................................................    1     1    Mm3
....................cuaGguacuagugccgcaggag......................................................................    3     1    Mm3
....................cuaaguacuagugccgcaggGg......................................................................    4     1    Mm3
....................cuaaguaUuagugccgcaggag......................................................................    2     1    Mm3
....................cuaaguacuagugccgcaggaC......................................................................    69    1    Mm3
....................cuaaguacuagugccgcGggag......................................................................    16    1    Mm3
```

Mature Star

```
augagagccaucgucagauacuaaguacuagugccgcaggaggaauauuaacucuccuaccguucugguacuuaagcuaggacauggcgaacacaucucuggagggagcuga
....................cuaaguacuagugccgcagAag......................................................................  3     1  Mm3
....................cuaaguGcuagugccgcaggag......................................................................  1     1  Mm3
....................cuaaguacuagugUcgcaggag......................................................................  2     1  Mm3
....................cuCaguacuagugccgcaggag......................................................................  1     1  Mm3
....................cAaaguacuagugccgcaggag......................................................................  5     1  Mm3
....................cuaaguacuagugccgcaggagA.....................................................................  180   1  Mm3
....................Nuaaguacuagugccgcaggagg.....................................................................  1     1  Mm3
....................cuaaguacuagugccgcaggagC.....................................................................  2     1  Mm3
....................cuaaguacuagugccgcaggagU.....................................................................  4034  1  Mm3
....................cuaaguacuagugccgcaggagg.....................................................................  76    0  Mm3
....................cuaaguacuagugccgcCggagg.....................................................................  1     1  Mm3
....................cuaaguacuagugccgcaggaggU....................................................................  16    1  Mm3
....................cuaaguacuagugccgcaggagAa....................................................................  3     1  Mm3
.....................uaaguacuagugccgcagg........................................................................  2     0  Mm3
.....................uaaguacuagugccgcagga.......................................................................  8     0  Mm3
.....................uaaguacuagugccgcaAga.......................................................................  1     1  Mm3
.....................uaaguacuagugccgcaggaC......................................................................  1     1  Mm3
.....................uaaguacuagugccgcaggag......................................................................  37    0  Mm3
.....................uaaguacuagAgccgcaggag......................................................................  1     1  Mm3
.....................uaaguacuagugccgcaAgag......................................................................  1     1  Mm3
.....................uaaguacuagugccgcaCgag......................................................................  1     1  Mm3
.....................uaaguacuagugccgcaUgag......................................................................  1     1  Mm3
.....................uaaguacuagugccgcaggaU......................................................................  1     1  Mm3
.....................uaaguacuagugccgcaggagU.....................................................................  20    1  Mm3
.....................uaaguacuagugccgcaggagg.....................................................................  4     0  Mm3
.....................uaaguacuagugccgcaggagC.....................................................................  1     1  Mm3
.....................uaaguacuagugccgcaggaggU....................................................................  1     1  Mm3
.......................................................ccuaccguucugguacuuaag....................................  1     0  Mm3

...................acuaaguacuagugccgcagga.......................................................................  10    0  Fm3
...................acuaaguacuagugccgcaggaU......................................................................  1     1  Fm3
....................cuaaguacuagugccgUa..........................................................................  2     1  Fm3
....................cuaaguacuagugccgca..........................................................................  11    0  Fm3
....................cuaaguacuagugccgcaA.........................................................................  3     1  Fm3
....................cuaaguacuagugccgcaU.........................................................................  7     1  Fm3
....................cuaaguacuagugccgcag.........................................................................  21    0  Fm3
....................cuaaguacuagugccgAagg........................................................................  1     1  Fm3
....................cuaaguacuagugccgcagg........................................................................  50    0  Fm3
....................cuaaguacuagugccgcagA........................................................................  1     1  Fm3
....................cuaaguacuagugccgcagU........................................................................  5     1  Fm3
....................cuaagNacuagugccgcagga.......................................................................  1     1  Fm3
....................cuaaguacuagugccgcUgga.......................................................................  4     1  Fm3
....................cuaaguacuagugccgcagUa.......................................................................  1     1  Fm3
....................cuaaguacuagugcUgcagga.......................................................................  1     1  Fm3
....................cuaaguacuagugccgcagga.......................................................................  1207  0  Fm3
....................cuaaguacuagugccgUagga.......................................................................  1     1  Fm3
....................Auaaguacuagugccgcagga.......................................................................  1     1  Fm3
....................cuaaguacuagugccgcGgga.......................................................................  4     1  Fm3
....................cAaaguacuagugccgcagga.......................................................................  1     1  Fm3
....................cuaaguacuagugccgcCgga.......................................................................  3     1  Fm3
....................cuaaguacuaCugccgcagga.......................................................................  1     1  Fm3
....................cuaaguacuagugccgAagga.......................................................................  11    1  Fm3
....................cuaUguacuagugccgcagga.......................................................................  1     1  Fm3
....................cCaaguacuagugccgcagga.......................................................................  1     1  Fm3
....................cuaagCacuagugccgcagga.......................................................................  1     1  Fm3
....................cuaaguacuaAugccgcagga.......................................................................  2     1  Fm3
....................cuaaguacuagugUcgcagga.......................................................................  4     1  Fm3
....................cuaaguacuagugccgcaggU.......................................................................  10    1  Fm3
....................cuaaguacuaUugccgcagga.......................................................................  2     1  Fm3
....................cuaaguacuagugccgGagga.......................................................................  2     1  Fm3
....................cuaaguacuGgugccgcagga.......................................................................  2     1  Fm3
....................cuCaguacuagugccgcaggag......................................................................  2     1  Fm3
....................cuaagCacuagugccgcaggag......................................................................  3     1  Fm3
....................cuaaguacuagugccgcagUag......................................................................  2     1  Fm3
....................cuaaguaGuagugccgcaggag......................................................................  1     1  Fm3
....................cGaaguacuagugccgcaggag......................................................................  2     1  Fm3
....................cuaagNacuagugccgcaggag......................................................................  4     1  Fm3
....................cuaagGacuagugccgcaggag......................................................................  1     1  Fm3
....................cuaaguacuagugccgcCggag......................................................................  27    1  Fm3
....................cuaaguacuagugccgcagAag......................................................................  1     1  Fm3
```

```
                    Mature                              Star
augagagccaucgucagauacuaaguacuagugccgcaggaggaauauuaacucuccuaccguucugguacuuaagcuaggacauggcgaacacaucucuggagggagcuga
....................cuGaguacuagugccgcaggag......................................................................    2     1    Fm3
....................cuaaguGcuagugccgcaggag......................................................................    1     1    Fm3
....................cuaaguacuagugccgcaggaC......................................................................    117   1    Fm3
....................cuaaguacuagugccgcUggag......................................................................    26    1    Fm3
....................cuaaguacuaCugccgcaggag......................................................................    6     1    Fm3
....................cuaaguacuaUugccgcaggag......................................................................    14    1    Fm3
....................cuaaguacuagugccgUaggag......................................................................    18    1    Fm3
....................cuaaguUcuagugccgcaggag......................................................................    1     1    Fm3
....................cuaaguacuagugccgcGggag......................................................................    23    1    Fm3
....................cuaaguacuaAugccgcaggag......................................................................    9     1    Fm3
....................cuaaguacuagugccgAaggag......................................................................    27    1    Fm3
....................cuaaguacuGgugccgcaggag......................................................................    1     1    Fm3
....................Uuaaguacuagugccgcaggag......................................................................    5     1    Fm3
....................cuaaguaUuagugccgcaggag......................................................................    1     1    Fm3
....................cuaaguacuagugccgcaggaA......................................................................    23    1    Fm3
....................cuUaguacuagugccgcaggag......................................................................    1     1    Fm3
....................cuaaguacuagugccUcaggag......................................................................    1     1    Fm3
....................Nuaaguacuagugccgcaggag......................................................................    6     1    Fm3
....................cuaaguacuagugccgcaggaU......................................................................    67    1    Fm3
....................cuaCguacuagugccgcaggag......................................................................    1     1    Fm3
....................cCaaguacuagugccgcaggag......................................................................    3     1    Fm3
....................cuaaguacuagugccgcaggUg......................................................................    1     1    Fm3
....................cuaaguacuagugccgcaNgag......................................................................    1     1    Fm3
....................cuaaguacuagugcUgcaggag......................................................................    5     1    Fm3
....................cuaaguacuagugAcgcaggag......................................................................    2     1    Fm3
....................cuaaguacuagugccgGaggag......................................................................    6     1    Fm3
....................Auaaguacuagugccgcaggag......................................................................    5     1    Fm3
....................cuaUguacuagugccgcaggag......................................................................    1     1    Fm3
....................cuaaguacuagugcGgcaggag......................................................................    1     1    Fm3
....................cuaaguacuagugccgcaggag......................................................................    7600  0    Fm3
....................cuaagAacuagugccgcaggag......................................................................    1     1    Fm3
....................cuaaguacuagugccgcaCgag......................................................................    2     1    Fm3
....................cuaaguacuagugccCcaggag......................................................................    1     1    Fm3
....................cAaaguacuagugccgcaggag......................................................................    4     1    Fm3
....................cuaaguacuagugUcgcaggag......................................................................    2     1    Fm3
....................cuaaguacuagugccgcaggagA.....................................................................    363   1    Fm3
....................cuaaguacuagugccgAaggagg.....................................................................    1     1    Fm3
....................cuaaguacuagugccgcaggagg.....................................................................    143   0    Fm3
....................cuaaguacuagugccgUaggagg.....................................................................    1     1    Fm3
....................cuaaguacuaAugccgcaggagg.....................................................................    1     1    Fm3
....................cuaaguacuagugccgcaggagC.....................................................................    1     1    Fm3
....................cuaaguacuagugccgcaggagU.....................................................................    2028  1    Fm3
....................cuGaguacuagugccgcaggagg.....................................................................    1     1    Fm3
....................cuaaguacuagugccgcCggagg.....................................................................    1     1    Fm3
....................cuaaguacuagugccgcaggagUa....................................................................    3     1    Fm3
....................cuaaguacuagugccgcaggaggU....................................................................    9     1    Fm3
....................cuaaguacuagugccgcaggagAa....................................................................    13    1    Fm3
....................cuaaguacuaguCccgcaggagga....................................................................    1     1    Fm3
.....................uaaguacuagugccgcag.........................................................................    3     0    Fm3
.....................uaaguacuagugccgcagg........................................................................    1     0    Fm3
.....................uaaguacuagugccgcagga.......................................................................    8     0    Fm3
.....................uaaguacuagugcGgcagga.......................................................................    1     1    Fm3
.....................uaaguacuagugccgcaggaC......................................................................    1     1    Fm3
.....................uaaguacuagugccgcaggCg......................................................................    1     1    Fm3
.....................uaaguacuagugccgcaggaU......................................................................    3     1    Fm3
.....................uaaguacuagugccgcCggag......................................................................    1     1    Fm3
.....................uaaguacuagugccgcaggag......................................................................    37    0    Fm3
.....................uaaguacuagugccgcaAgagg.....................................................................    1     1    Fm3
.....................uaaguacuagugccgcaggagg.....................................................................    7     0    Fm3
.....................uaaguacuagugccgcaggagU.....................................................................    13    1    Fm3
.....................uGaguacuagugccgcaggagg.....................................................................    3     1    Fm3
.....................uaaguacuagugccgcaggagga....................................................................    5     0    Fm3
........................guacuagugccgcaggag......................................................................    2     0    Fm3
.......................................................ccuaccguucugguacuuaagc...................................    5     0    Fm3
.......................................................ccuaccguuAugguacuuaagc...................................    1     1    Fm3
........................................................cuaccguucugguacuuaagcu..................................    3     0    Fm3
```
